# Robot-assisted extended thymectomy in juvenile myasthenia gravis: first case performed in Ecuador

**DOI:** 10.1093/jscr/rjaf759

**Published:** 2025-10-18

**Authors:** Enrique Gagliardo, Luis Aguaguiña Toainga, Janneth Alejandra Pérez Cedeño, Luis Alberto Barrera Delgado, Luis Munoz Andrade

**Affiliations:** National Oncology Institute SOLCA Guayaquil, Department of Thoracic Surgery, Guayaquil, Guayas 090505, Ecuador; Hospital General Monte Sinaí, Guayaquil, Ecuador; National Oncology Institute SOLCA Guayaquil, Department of Thoracic Surgery, Guayaquil, Guayas 090505, Ecuador; National Oncology Institute SOLCA Guayaquil, Department of Thoracic Surgery, Guayaquil, Guayas 090505, Ecuador; National Oncology Institute SOLCA Guayaquil, Department of Thoracic Surgery, Guayaquil, Guayas 090505, Ecuador

**Keywords:** myasthenia gravis, robotic thymectomy, juvenile MG, Da Vinci system, Ecuador, autoimmune neuromuscular disorder

## Abstract

Myasthenia gravis (MG) is a chronic autoimmune disorder characterized by fatigable muscle weakness, typically caused by antibodies against acetylcholine receptors (AChR) or muscle-specific kinas. While it primarily affects adults, juvenile cases represent a significant minority and often pose greater therapeutic challenges. Extended thymectomy has proven beneficial in AChR-positive MG, reducing symptoms and immunosuppressive needs. Robotic-assisted thymectomy offers enhanced precision with lower morbidity. We report the first such procedure in Ecuador, performed on a 21-year-old woman with recurrent myasthenic crises. The surgery was uneventful, and follow-up showed marked clinical improvement without recurrence, highlighting the feasibility of advanced techniques in low-resource settings.

## Introduction

Myasthenia gravis (MG) is a rare autoimmune disorder of the neuromuscular junction, caused by autoantibodies against the acetylcholine receptor (AChR) or, less commonly, muscle-specific tyrosine kinase [1]. It typically presents with fluctuating weakness of voluntary muscles that worsens with exertion and improves with rest. Juvenile MG, defined by onset before age 18, comprises 10%–15% of cases and affects females more frequently [2].

Thymectomy has long been used to treat MG, based on its central role in disease pathogenesis via ectopic germinal center formation and autoantibody production [3]. The Myasthenia Gravis Thymectomy Trial (MGTX) trial confirmed that extended thymectomy improves clinical outcomes and reduces corticosteroid dependency in AChR-positive patients [4].

Robotic-assisted surgery has further enhanced this technique, providing 3D visualization, articulated instruments, and improved precision for perithymic dissection [5]—advantages that are particularly valuable in complex mediastinal anatomy [6].

Although robotic thymectomy is increasingly common worldwide, its use remains limited in low- and middle-income countries due to cost and infrastructure. We report the first case of robot-assisted extended thymectomy in Ecuador, demonstrating the feasibility of introducing advanced surgical techniques in resource-constrained settings and their clinical value in managing juvenile MG.

## Case report

A 21-year-old female with a past medical history of idiopathic thrombocytopenic purpura and autoimmune hepatitis presented with generalized muscle weakness, ptosis, dysphagia, and progressive dyspnea. She had been diagnosed with seropositive generalized MG and experienced four documented myasthenic crises within the previous year.

Neurological examination revealed fatigable dysphonia, bilateral ptosis, and muscle strength graded 4/5 on the Medical Research Council scale. Laboratory testing confirmed the presence of AChR antibodies and positive smooth muscle antibody at a titer of 1:20.

**Figure 1:**
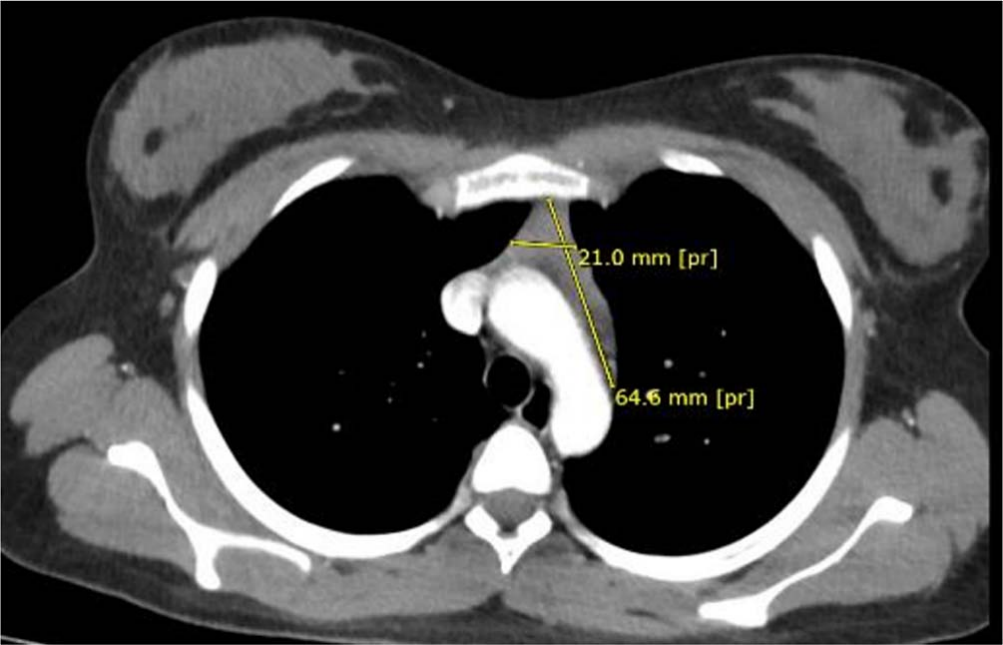
Preoperative thoracic CT showing a 61 × 24 mm homogeneous anterior mediastinal mass.

High-resolution chest computed tomography (CT) demonstrated a homogeneous anterior mediastinal mass measuring 61 × 24 mm, consistent with thymic hyperplasia (Fig. 1).

**Figure 2:**
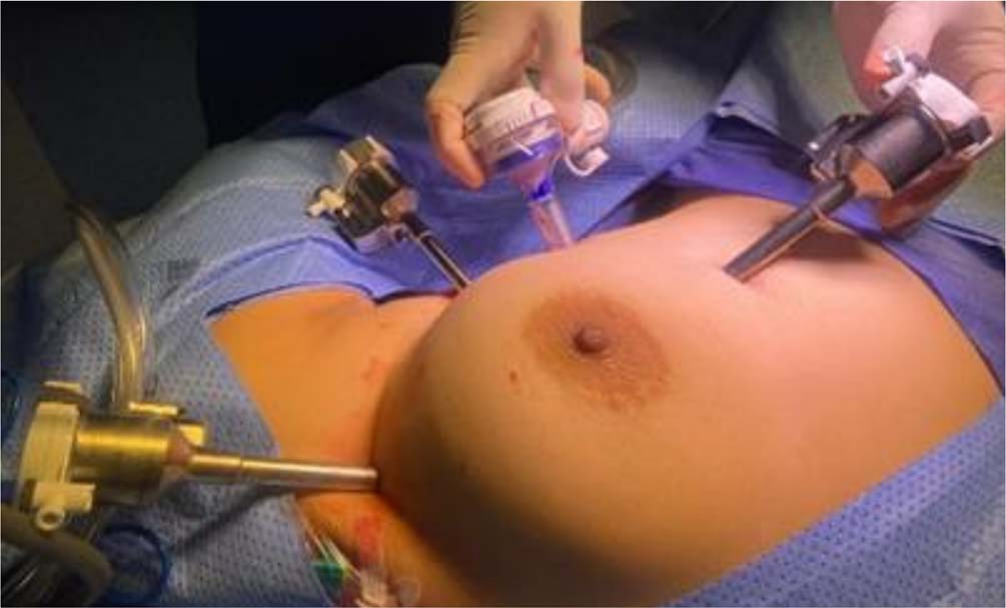
Port placement for robotic-assisted thymectomy using the Da Vinci Xi system.

A multidisciplinary team recommended extended thymectomy. The procedure was performed using the Da Vinci Xi robotic system through a left-sided thoracoscopic approach. Three 8 mm robotic ports and one assistant port were placed (Fig. 2). General anaesthesia was used, and no epidural or nerve block was applied. Single-lung ventilation was performed using a right-sided selective tube to allow optimal exposure for the left-sided robotic docking. A surgical pneumothorax was created with CO₂ insufflation at 5 mmHg pressure and 7 L/min flow to provide adequate working space.

**Figure 3:**
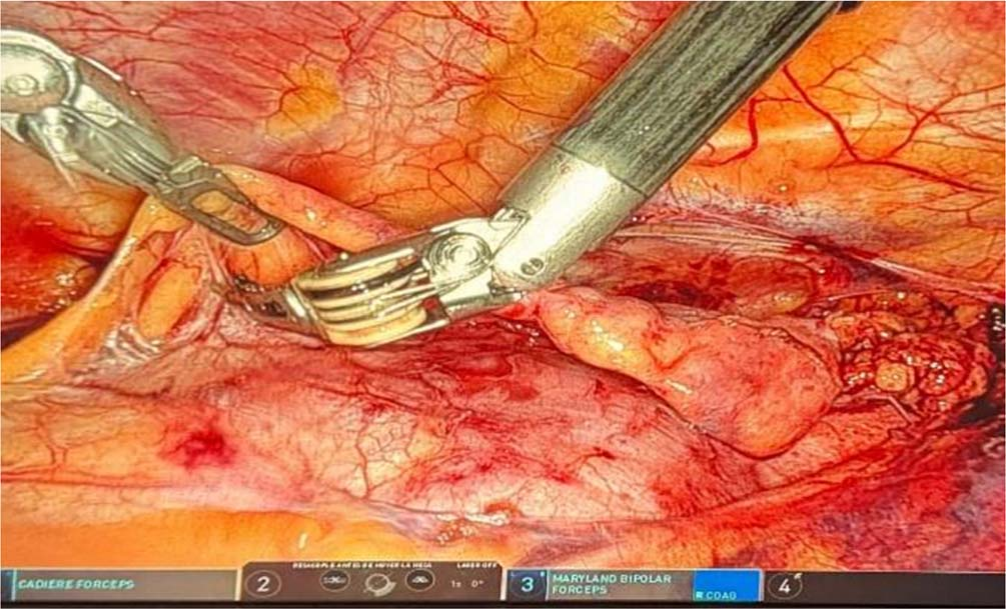
Intraoperative robotic dissection of the inferior thymic pole.

Dissection extended from the diaphragm inferiorly to the left and right phrenic nerves laterally and up to the innominate vein superiorly. The thymectomy was complete, including perithymic and pre-cardiac fat. Confirmation of the right phrenic nerve was mandatory, and resection extended to this structure to ensure radical removal. The thymus and all surrounding mediastinal fat were removed (Fig. 3). The operative time was 155 minutes with minimal blood loss.

Postoperative recovery was uneventful. The patient was discharged on postoperative Day 4. At 2 weeks and 3 months follow-up, she reported complete resolution of ptosis, absence of dysphonia, improved muscle strength, and no recurrence of myasthenic symptoms.

## Results

The robotic-assisted extended thymectomy was completed without intraoperative complications. The use of general anesthesia with right-sided single-lung ventilation and controlled CO₂ insufflation (5 mmHg, 7 L/min) provided optimal mediastinal exposure, allowing a complete resection between both phrenic nerves without complications. Using a left thoracoscopic approach and the Da Vinci Xi system, the surgical team achieved optimal exposure of the anterior mediastinum, enabling precise dissection of thymic and perithymic tissues. The procedure lasted 155 minutes with minimal blood loss (<50 ml), and no conversion to open surgery or transfusions were required.

Postoperative recovery was smooth. The patient was extubated immediately, resumed oral intake within 24 hours, and ambulated independently by Day 2. She was discharged on postoperative Day 4 with complete resolution of dysphonia, improved palpebral function, and full muscle strength (Daniels 5/5).

At three-month follow-up, she remained asymptomatic with no further myasthenic crises. Corticosteroids were successfully tapered, and she reported improved functional performance in daily activities. Histopathology confirmed thymic hyperplasia without malignancy, and complete resection—including mediastinal fat between both phrenic nerves and up to the innominate vein—was verified intraoperatively.

**Figure 4:**
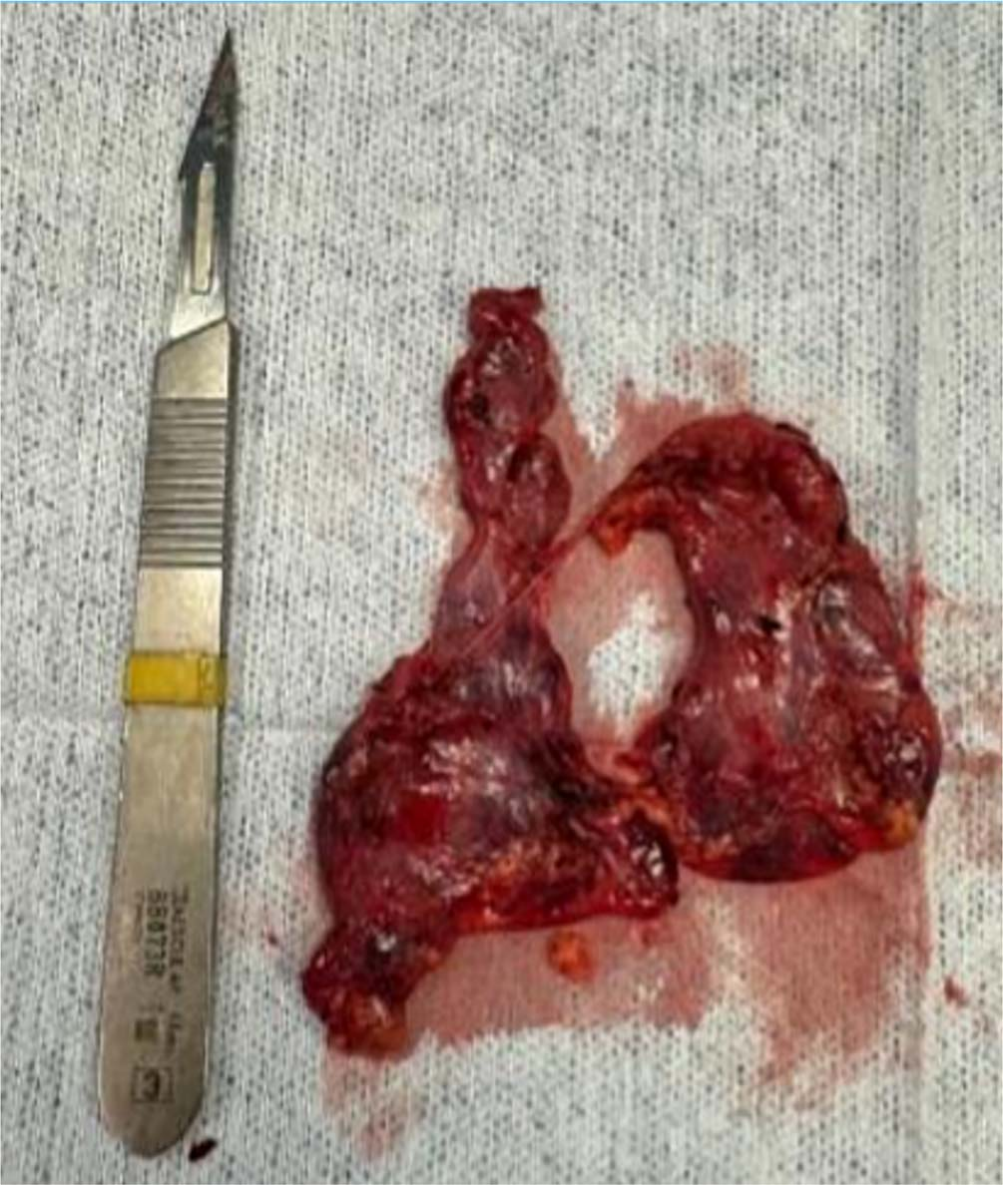
Resected thymic tissue with surrounding mediastinal fat.

This case illustrates the safety, efficacy, and neurological benefit of robotic-assisted thymectomy in juvenile MG, and highlights the viability of advanced surgical approaches in a Latin American setting (Fig. 4).

## Discussion

The benefits of thymectomy in MG have been supported by several studies. The MGTX trial provided level 1 evidence of improved clinical status and reduced immunosuppressive use in AChR-positive patients undergoing extended thymectomy [4]. Subsequent analyses suggest that more extensive resections—including the perithymic fat and ectopic thymic tissue—may contribute to better outcomes, reducing the risk of disease recurrence [7].

Robotic-assisted thymectomy has increasingly become the preferred approach in high-volume centers due to its superior visualization, dexterity, and precision, which facilitate complete resection of mediastinal tissue while avoiding injury to adjacent structures [5, 6]. Compared to open or video-assisted thoracoscopic surgery approaches, robotic surgery is associated with lower morbidity, shorter hospital stays, and faster return to baseline function [8].

In Ecuador and many developing countries, access to robotic surgery remains limited by infrastructure and cost. This case represents a milestone in regional surgical practice, demonstrating that even in resource-limited environments, robotic thymectomy can be safely and effectively implemented with proper training and institutional commitment.

Furthermore, this case highlights the potential for robotic surgery to transform outcomes in chronic autoimmune diseases such as MG, which impose a substantial burden on quality of life, particularly in young adults. Early surgical intervention in juvenile MG may modify the course of disease, reduce long-term dependence on corticosteroids, and prevent progression to refractory stages [3].

## Conclusion

Robotic-assisted extended thymectomy offers a safe, precise, and effective surgical option for patients with generalized MG, even in settings with limited resources. This case, the first of its kind in Ecuador, confirms that advanced surgical technologies can be adopted successfully in developing countries to improve clinical outcomes. The substantial improvement in the patient’s neuromuscular function supports the early use of extended thymectomy as part of the treatment strategy in juvenile AChR-positive MG.
